# Assessment of WHO criteria for identifying ART treatment failure in Vietnam from 2007 to 2011

**DOI:** 10.1371/journal.pone.0182688

**Published:** 2017-09-06

**Authors:** Nicole K. Le, Emilia Riggi, Gaetano Marrone, Tam Van Vu, Ricardo O. Izurieta, Chuc Kim Thi Nguyen, Mattias Larsson, Cuong Duy Do

**Affiliations:** 1 Morsani College of Medicine, University of South Florida, Tampa, FL, United States of America; 2 Department of Brain and Behavioural Sciences, Medical Statistics Unit, University of Pavia, Pavia, Italy; 3 Department of Public Health Sciences, Karolinska Institutet, Stockholm, Sweden; 4 Department of Infectious Diseases, Uong Bi General Hospital, Uong Bi, Quang Ninh, Vietnam; 5 Department of Global Health, College of Public Health, University of South Florida, Tampa, FL, United States of America; 6 Hanoi Medical University, Hanoi, Vietnam; 7 Infectious Diseases Department, Bach Mai Hospital, Hanoi, Vietnam; Azienda Ospedaliera Universitaria di Perugia, ITALY

## Abstract

**Objective:**

We evaluated the sensitivity and specificity of the WHO immunological criteria for detecting antiretroviral therapy (ART) treatment failure in a cohort of Vietnamese patients. We conducted a stratified analysis to determine the effects of BMI, peer support, adherence to antiretroviral (ARV) drugs, age, and gender on the sensitivity and specificity of the WHO criteria.

**Methods:**

We conducted a retrospective cohort study of 605 HIV-infected patients using data previously collected from a cluster randomized control trial study. We compared the sensitivity and specificity of CD4^+^ counts to the gold standard of virologic testing as a diagnostic test for ART failure at different time points of 12, 18, and 24 months.

**Results:**

The sensitivity [95% confidence interval (CI)] of the WHO immunological criteria based on a viral load ≥ 1000 copies/mL was 12% (5%-23%), 14% (2%-43%), and 12.5% (2%-38%) at 12, 18, and 24 months, respectively. In the same order, the specificity was 93% (90%-96%), 98% (96%-99%), and 98% (96%-100%). The positive predictive values (PPV) at 12, 18, and 24 months were 22% (9%-40%), 20% (3%-56%), and 29% (4%-71%); the negative predictive values (NPV) at the same time points were 87% (84%-90%), 97% (95%-98%), and 96% (93%-98%). The stratified analysis revealed similar sensitivities and specificities.

**Conclusion:**

The sensitivity of the WHO immunological criteria is poor, but the specificity is high. Although testing costs may increase, we recommend that Vietnam and other similar settings adopt viral load testing as the principal method for determining ART failure.

## Introduction

Surveillance of HIV antiretroviral therapy failure has been challenging in resource-limited settings. This has resulted in suboptimal identification of treatment failure [1]. In settings lacking support for the gold standard of routine viral load (VL) monitoring, countries have adopted the World Health Organization (WHO) clinical and immunological criteria for detection of treatment failure [2–5]. These guidelines define clinical treatment failure as occurrence or recurrence of stage 4 diseases or conditions after at least 6 months of therapy.

Early identification of ART treatment failure allows patients a higher chance of success when switching to a second line ART [6]. Mounting evidence has shown that the WHO criteria for ART monitoring has poor sensitivity and specificity for detecting treatment failure, especially for higher baseline CD4^+^ cell counts, when compared to the gold standard of VL monitoring [7–14]. (The gold standard is the recommended conventional method of diagnosing a particular disease, or in this case, ART treatment failure. Any new test needs to be compared against the gold standard. The information obtained by comparing a new diagnostic test with the gold standard is conventionally summarized in a two-by-two table.) The Vietnam Guidelines define virologic failure as plasma VL > 5,000 copies/mL, while the WHO virologic criteria defines it as plasma VL ≥ 1,000 copies/mL [15, 16].

In the past 15 years, Vietnam has increased its investment in HIV prevention, care, and treatment with the support of international aid agencies. This effort has been mainly targeted at high risk populations, which include people who inject drugs [17]. The national prevalence of injecting drug use, the leading mode of HIV transmission in Vietnam, has been decreasing from 26% in 2011 to an estimate of 23% in 2015 [17]. Despite this progress, high HIV prevalence among people who inject drugs persists in some cities and provinces, such as Quang Ninh (56% in 2013), where our study took place [17, 18].

On October 25, 2014, Vietnam became the first country in Asia to commit to expanding HIV treatment by adopting the UNAIDS 90-90-90 targets [19]. This aims to have 90% of all people living with HIV to be aware of their HIV status, 90% of all people with diagnosed HIV infection to receive sustained antiretroviral therapy, and 90% of all people receiving antiretroviral therapy to have viral suppression by 2020 [20].

The Vietnam Ministry of Health (MOH) adopted the WHO clinical and immunological criteria, described previously, for their guidelines. Despite the addition of routine VL testing every 6 months, the test is only performed in a few select laboratories in large cities like Ha Noi and Ho Chi Minh City [4, 21, 22]. Furthermore, public international programs may not support routine VL monitoring unless patients meet the WHO clinical and immunological treatment failure criteria [23]. Even with this targeted VL strategy for confirming suspected treatment failure, this approach still has the potential to delay treatment switching [16].

This study sought to determine the sensitivity and specificity of the WHO immunological criteria for identifying ART treatment failure in resource-limited settings. As VL testing is not routinely done in Vietnam, there isn’t much published data on the effectiveness of the Vietnam National Guidelines [23]. This study also investigated the effects of BMI, peer support, adherence to ARV, age, and gender on the sensitivity and specificity of the WHO immunological criteria.

## Methods

This retrospective cohort study collected information from HIV-infected patients on first-line ART from a cluster randomized controlled trial carried out in a rural resource-limited setting of Quang Ninh, Vietnam between July 2007 and November 2011 [24]. The inclusion criteria for the 605 patients of this study was ART-naïve HIV-infected patients. Data extracted from a 24 month follow up included CD4^+^ levels, viral load levels, adherence to ARV, gender, BMI, peer support, and age.

### Measures

For the purposes of this analysis, WHO immunologic failure was diagnosed if the participant met one of the following criteria:

1. CD4^+^ counts that return to or fall below pre-therapy baseline level,
2. 50% decline of CD4^+^ from the on-treatment peak value after at least 6 months of the initiation of ART,
3. CD4^+^ count <100 cells/μL after a year without any increase [3, 15].

The current Vietnam guidelines for viral load defines treatment failure at viral load > 5000 copies/mL [15]. However, the current WHO guidelines define treatment failure at VL ≥ 1000 copies/mL [16]. Data were analyzed using these two different virologic failure thresholds as the gold standard.

### Statistical analysis

We presented descriptive continuous data as median and interquartile range (IQR) and listed categorical variables as numbers and percentages. We determined the sensitivity and specificity for predicting various definitions of virologic failure mentioned previously at 12, 18, and 24 months after initiation of ART. We determined the positive and negative predictive values of the immunologic failure criteria as well. In addition, we adjusted the diagnostic test analysis with both VL > 5000 copies/mL and VL ≥ 1000 copies/mL, for several variables such as gender, age, BMI, peer support, and adherence to ARV. The results are presented in the tables with the corresponding 95% confidence interval (CI). The confidence intervals were based on formulae provided by Simel et al [25].

BMI was stratified between below 18 kg/m^2^ and above 18 kg/m^2^. Patients were stratified into groups that have or don’t have peer support. Peer support involved home-based adherence counseling by fellow HIV-infected peer supporters [24]. Adherence to ARV was stratified into no missed doses and one or more missed doses. Age was split between above and below 32 years (the median age of patients in this study was 31.90 years), while gender was divided into male and female. The analysis was carried out using R software [26]. The package used to compute the confidence intervals was “epiR” [27].

### Ethical considerations

The study was approved by the Institutional Review Boards of Hanoi Medical University, Ministry of Health, Vietnam (numbers 26/IRB, 66/HMURB, 59/HMURB, and 98/HMURB), the Regional Board for Ethics Review from Karolinska Institutet in Stockholm, Sweden (number 2006/1367-31/4), and the Institutional Review Board (no. Pro00027277) at the University of South Florida.

## Results

This study included the baseline characteristics of a total of 605 HIV-positive patients (Table 1). These patients ranged from 20 to 56 years of age.

**Table 1:** Baseline characteristics of the study sample.

Variable	Number (%) (N = 605)^a^	
**Gender**		
▪ Male	425 (70)	
▪ Female	180 (30)	
**BMI**		
• <18 kg/m^2^	217 (35.9%)	
• >18 kg/m^2^	388 (64.1%)	
**Education**		
• Illiterate/primary school	72 (11.9)	
• Middle school	236 (39)	
• High school	240 (39.7)	
• University	57 (9.4)	
**Employment**		
• Employed	474 (78.5)	
• Unemployed	130 (21.5)	
**Heroin use**		
• Yes	314 (51.9)	
• No	291 (48.1)	
**Family member with HIV**		
• Yes	247 (40.8)	
• No	358 (59.2)	
	**Median (Na%)**	**IQR**
**Age (years)**	31.90	29.29–35.16
**Weight (Kg)**	50 (14)	45–55
**CD4^+^ at start of ART (cells/μL)**	84 (1)	29–177
**VL at start of ART (copies/mL)**	50115	10702–147627

^**a**^ The listed categorical variables are represented as numbers and percentages, while continuous data as median and interquartile range (IQR).

Na is the % of missing data for that variable.

**Fig 1:**
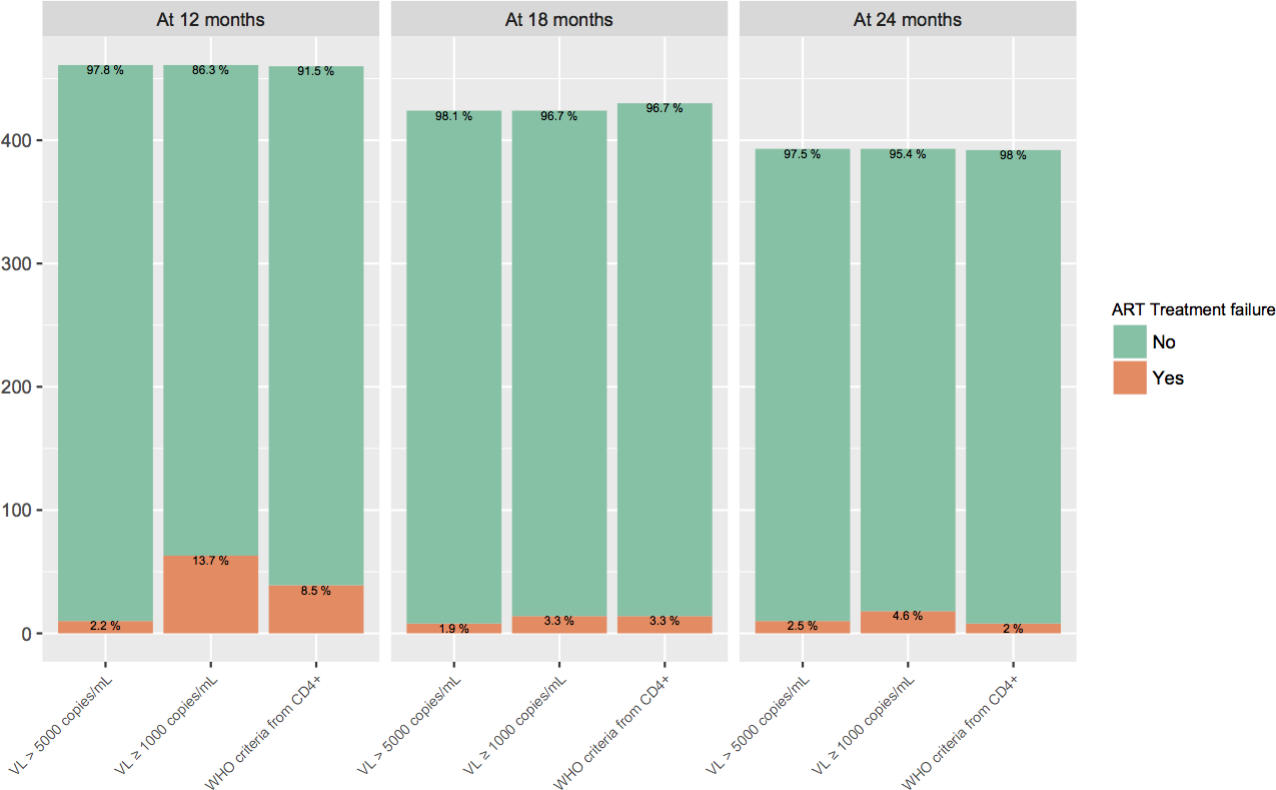
ART treatment failure identification from different definitions. The viral load definitions were considered as gold standard, and the WHO criteria from CD4^+^ as the new test.

Fig 1 represents the frequency of ART treatment failure in our sample based on virologic criteria (VL > 5000 copies/mL and VL ≥ 1000 copies/mL) and WHO immunological criteria (CD4^+^).

Among the different definitions, the proportions of ART treatment failure were less than the proportions of NO ART treatment failure. The virologic criterion VL ≥ 1000 copies/mL got the highest proportion of ART treatment failure at different times.

### Diagnostic test analysis

**Fig 2:**
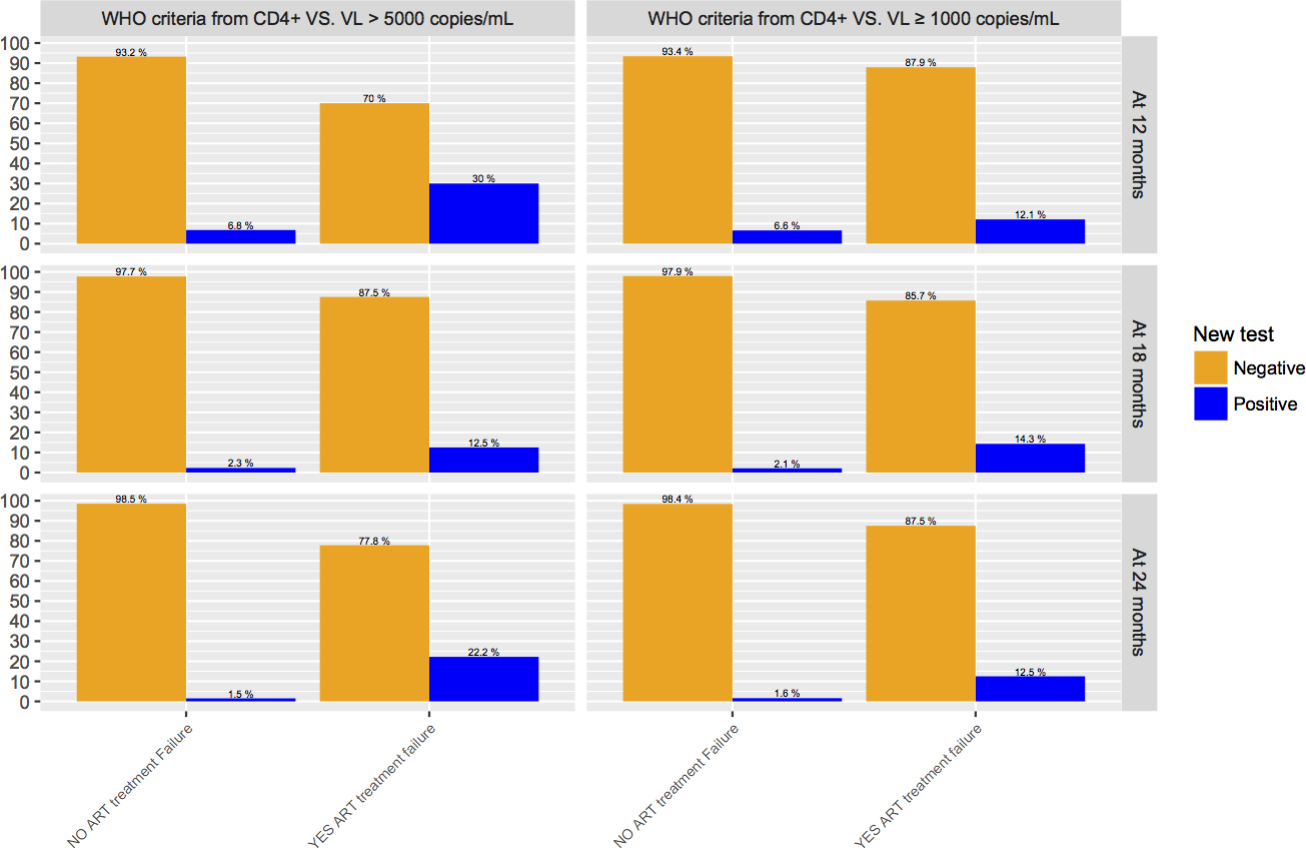
Comparison between the gold standard tests and the CD4^+^ test at the 12th, 18th, and 24th months.

As shown in Fig 2, all of this information was collected based on treatment failure defined by the Vietnam guidelines (VL > 5000 copies/mL) and WHO Guidelines (VL ≥ 1000 copies/mL), both considered as gold standards, with the overall WHO immunological criteria, 12, 18, and 24 months after the start of treatment.

### Vietnam guidelines

The sensitivity, based on treatment failure at viral load > 5000 copies/mL and the overall WHO immunological criteria 12 months after the start of treatment, was 30% and the specificity was 93%. However, among the people who tested positive for WHO immunological criteria, only 9% actually had treatment failure (the corresponding PPV). For those that tested negative, 98% did not have the treatment failure (NPV).

At 18 months, the sensitivity and specificity were 12.5% and 98%, respectively, while the PPV and NPV were 10% and 98%, respectively.

On the contrary, at 24 months after treatment initiation, the sensitivity was 22%. The PPV, among patients that tested positive, was 29% that had ART treatment failure. All the indexes are reported in Table 2.

**Table 2:** Diagnostic test indexes of the gold standard test (VL > 5000 copies/mL and VL ≥ 1000 copies/mL) and the CD4^+^ test at 12, 18, and 24 months. The absolute estimates with their 95% CIs are also shown.

	VL > 5000 copies/mL comparing WHO criteria from CD4^+^			
	Sensitivity	Specificity	Positive predictive value	Negative predictive value
At 12^th^ month	0.300	0.932	0.094	0.983
	[0.067–0.652]	[0.903–0.954]	[0.020–0.250]	[0.965–0.993]
	30%	93%	9%	98%
	[7–65%]	[90–95%]	[2–25%]	[97–99%]
At 18^th^ month	0.125	0.977	0.100	0.982
	[0.003–0.527]	[0.956–0.989]	[0.003–0.445]	[0.963–0.993]
	12.5%	98%	10%	98%
	[0.3–53%]	[96–99%]	[0.3–45%]	[96–99%]
At 24^th^ month	0.222	0.985	0.286	0.978
	[0.028–0.600]	[0.964–0.995]	[0.037–0.710]	[0.956–0.991]
	22%	99%	29%	98%
	[3–60%]	[96–100%]	[4–71%]	[96–99%]
	**VL ≥ 1000 copies/mL comparing WHO criteria from CD4**^**+**^			
At 12^th^ month	0.121	0.934	0.219	0.873
	[0.050–0.233]	[0.904–0.957]	[0.093–0.400]	[0.837–0.904]
	12%	93%	22%	87%
	[5–23%]	[90–96%]	[9–40%]	[84–90%]
At 18^th^ month	0.143	0.979	0.200	0.969
	[0.018–0.428]	[0.959–0.991]	[0.025–0.556]	[0.946–0.984]
	14%	98%	20%	97%
	[2–43%]	[96–99%]	[3–56%]	[95–98%]
At 24^th^ month	0.125	0.984	0.286	0.957
	[0.016–0.383]	[0.963–0.995]	[0.037–0.710]	[0.929–0.976]
	12.5%	98%	29%	96%
	[2–38%]	[96–100%]	[4–71%]	[93–98%]

### WHO guidelines

Moving to the two-by-two table between WHO immunological criteria and VL ≥ 1000 copies/mL, the sensitivity indexes were lower compared to those mentioned previously at the 12^th^ and 24^th^ months, while at 18 months, it was slightly increased (Table 2). The specificity indexes were the same, except for at 24 months after the start of treatment.

As previously mentioned, the diagnostic test analysis was stratified at three different times for several variables including gender, age, BMI, peer support, and adherence to ARV. We summarized the results comparing the two gold standards and the CD4^+^ test with detecting ART treatment failure in Table 3, using the different strata. Similar to the results in Table 2, the sensitivities ranged from 0–50% and the specificities ranged from 92–100%.

**Table 3:** Diagnostic test analysis of the gold standard test (at both the VL cutoffs of 5000 and 1000 copies/mL) and the CD4^+^ test adjusted for confounders at 12, 18, and 24 months. Results are shown as point estimates and [95% CI].

	**VL > 5000 copies/mL comparing WHO criteria from CD4^+^**								
	**Male**				**Female**				
	**Sensitivity**	**Specificity**	**PPV**	**NPV**	**Sensitivity**		**Specificity**	**PPV**	**NPV**
**At 12**^**th**^ **month**	25%	92%	8%	98%	50%		96%	12.5%	99%
	[3–65%]	[88–95%]	[1–27%]	[95–99%]	[1–99%]		[91–98%]	[0.3–53%]	[96–100%]
**At 18**^**th**^ **month**	14%	97%	11%	98%	0%		99%	0%	99%
	[0.4–58%]	[94–99%]	[0.3–48%]	[95–99%]	[0–99%]		[96–100%]	[0–99%]	[96–100%]
**At 24**^**th**^ **month**	14%	98%	25%	97%	50%		99%	33%	99%
	[0.4–58%]	[96–100%]	[0.6–81%]	[93–99%]	[1–99%]		[95–100%]	[0.8–91%]	[96–100%]
	**BMI < 18 kg/m**^**2**^				**BMI ≥ 18 kg/m**^**2**^				
	**Sensitivity**	**Specificity**	**PPV**	**NPV**	**Sensitivity**		**Specificity**	**PPV**	**NPV**
**At 12**^**th**^ **month**	25%	93%	9%	98%	33%		93%	10%	99%
	[0.6–81%]	[87–97%]	[0.2–41%]	[94–100%]	[4–78%]		[90–96%]	[1–30%]	[96–100%]
**At 18**^**th**^ **month**	0%	98%	0%	98%	17%		97%	13%	98%
	[0–91%]	[94–100%]	[0–91%]	[94–100%]	[0.4–64%]		[95–99%]	[0.3–53%]	[96–99%]
**At 24**^**th**^ **month**	0%	99%	0%	97%	33%		98%	33%	98%
	[0–81%]	[95–100%]	[0–99%]	[92–99%]	[4–78%]		[95–100%]	[4–78%]	[95–100%]
	**No peer support**				**Yes peer support**				
	**Sensitivity**	**Specificity**	**PPV**	**NPV**	**Sensitivity**	**Specificity**		**PPV**	**NPV**
**At 12**^**th**^ **month**	33%	95%	15%	98%	25%	92%		5%	99%
	[4–78%]	[90–97%]	[2–45%]	[95–99%]	[0.6–81%]	[8–95%]		[0.1–26%]	[96–100%]
**At 18**^**th**^ **month**	33%	98%	25%	99%	0%	97%		0%	98%
	[0.8–91%]	[95–100%]	[0.6–81%]	[96–100%]	[0–64%]	[94–99%]		[0–58%]	[94–99%]
**At 24**^**th**^ **month**	0%	98%	0%	97%	50%	99%		50%	99%
	[0–64%]	[94–100%]	[0–81%]	[93–99%]	[7–93%]	[96–100%]		[7–93%]	[96–100%]
	**Missed doses ≥ 1**				**No missed doses**				
	**Sensitivity**	**Specificity**	**PPV**	**NPV**	**Sensitivity**	**Specificity**		**PPV**	**NPV**
**At 12**^**th**^ **month**	33%	94%	13%	98%	25%	92%		6%	98%
	[4–78%]	[90–97%]	[2–38%]	[96–100%]	[0.6–81%]	[87–95%]		[0.2–30%]	[95–100%]
**At 18**^**th**^ **month**	25%	99%	25%	99%	0%	96%		0%	98%
	[0.6–81%]	[96–100%]	[0.6–81%]	[96–100%]	[0–72%]	[92–99%]		[0–58%]	[94–99%]
**At 24**^**th**^ **month**	0%	99%	0%	99%	33%	98%		40%	97%
	[0–81%]	[97–100%]	[0–91%]	[96–100%]	[4–78%]	[93–00%]		[5–85%]	[92–99%]
	**VL ≥ 1000 copies/mL comparing WHO criteria from CD4**^**+**^								
	**Male**				**Female**				
	**Sensitivity**	**Specificity**	**PPV**	**NPV**	**Sensitivity**	**Specificity**		**PPV**	**NPV**
**At 12**^**th**^ **month**	15%	92%	25%	87%	6%	95%		13%	89%
	[6–30%]	[88–95%]	[10–47%]	[82–91%]	[0.1–27%]	[90–98%]		[0.3–53%]	[83–93%]
**At 18**^**th**^ **month**	9%	96%	11%	96%	33%	100%		100%	99%
	[0.2–41%]	[93–99%]	[0.3–48%]	[92–98%]	[0.8–90%]	[96–100%]		[1–100%]	[95–100%]
**At 24**^**th**^ **month**	7%	98%	25%	94%	33%	99%		33%	99%
	[0.2–36%]	[95–100%]	[0.6–80%]	[90–97%]	[0.8–91%]	[95–100%]		[0.8–91%]	[95–100%]
	**BMI < 18 kg/m**^**2**^				**BMI ≥ 18 kg/m**^**2**^				
	**Sensitivity**	**Specificity**	**PPV**	**NPV**	**Sensitivity**	**Specificity**		**PPV**	**NPV**
**At 12**^**th**^ **month**	7%	92%	18%	80%	17%	94%		24%	91%
	[0.9–24%]	[86–96%]	[2–52%]	[73–87%]	[6–35%]	[90–97%]		[9–47%]	[87–94%]
**At 18**^**th**^ **month**	0%	98%	0%	96%	22%	98%		25%	97%
	[0–64%]	[94–100%]	[0–91%]	[91–100%]	[3–60%]	[95–99%]		[3–65%]	[95–99%]
**At 24**^**th**^ **month**	0%	99%	0%	94%	20%	98%		33%	96%
	[0–58%]	[95–100%]	[0–99%]	[88–98%]	[3–57%]	[95–100%]		[5–77%]	[93–98%]
	**No peer support**				**Yes peer support**				
	**Sensitivity**	**Specificity**	**PPV**	**NPV**	**Sensitivity**	**Specificity**		**PPV**	**NPV**
**At 12**^**th**^ **month**	11%	94%	23%	88%	13%	92%		21%	87%
	[2–29%]	[90–97%]	[5–54%]	[82–92%]	[4–30%]	[88–96%]		[6–46%]	[82–91%]
**At 18**^**th**^ **month**	17%	98%	25%	97%	13%	97%		17%	97%
	[0.4–64%]	[95–100%]	[0.6–81%]	[94–99%]	[0.3–53%]	[94–99%]		[0.4–64%]	[93–99%]
**At 24**^**th**^ **month**	0%	98%	0%	95%	25%	99%		50%	97%
	[0–48%]	[94–100%]	[0–81%]	[90–98%]	[3–65%]	[96–100%]		[7–93%]	[93–99%]
	**Missed doses ≥ 1**				**No missed doses**				
	**Sensitivity**	**Specificity**	**PPV**	**NPV**	**Sensitivity**	**Specificity**		**PPV**	**NPV**
**At 12**^**th**^ **month**	9%	94%	25%	82%	23%	93%		19%	94%
	[3–21%]	[90–97%]	[7–52%]	[77–87%]	[5–54%]	[88–96%]		[4–46%]	[90–97%]
**At 18**^**th**^ **month**	13%	99%	25%	97%	17%	97%		17%	97%
	[0.3–53%]	[96–100%]	[0.6–81%]	[94–99%]	[0.4–64%]	[93–99%]		[0.4–64%]	[93–99%]
**At 24**^**th**^ **month**	0%	99%	0%	96%	25%	98%		40%	95%
	[0–48%]	[96–100%]	[0–91%]	[92–98%]	[3–65%]	[93–100%]		[5–85%]	[90–98%]

## Discussion

We found that the WHO immunological criteria have a very low sensitivity and high specificity. The stratified analysis also didn't obtain results in favor of the CD4^+^ test. Due to low sensitivity of the criteria, it was not possible to accurately detect treatment failure. Therefore, the CD4^+^ diagnostic test is poor for detecting ART failure, and patients’ immune competence would have declined unnoticed as they progressed faster towards clinical failure and AIDS. This indicates that the WHO immunological criteria has too low a sensitivity to be used as a first line screening method. Based on this, we recommend for the WHO to change the treatment failure guidelines to be based solely on viral load in resource limited settings.

To the best of our knowledge, this is the first study to report the sensitivities and specificities of the WHO immunological criteria compared to the gold standard of viral load testing in Vietnam. Some countries have a targeted approach to viral load testing (e.g. Cambodia, India, and Vietnam) where patients are only tested if treatment failure is suspected using WHO clinical and immunological criteria [15, 28, 29]. Despite being less expensive than routine testing in the short term, this approach risks delaying treatment failure identification [16]. With earlier identification of treatment failure and earlier interventions to improve adherence, the more timely switch to second line ART could decrease the immunological detrition as well as prevent accumulation of resistance mutations [4, 30]. This would decrease the risk of disease progression, ARV drug resistance, and further HIV transmission [16]. In the long run, it may be more cost effective to reduce these incidences through a more robust test for treatment failure, as delaying its identification can have high long-term costs including more expensive second-line drug regimens and an increased risk of transmitting drug resistant HIV strains.

Viral load testing accurately and precisely identifies treatment failure as well as non-adherence [4]. Such an approach would prevent misdiagnosis of treatment failure and avoid the unnecessary change to a more expensive second line regimen [10, 11, 13]. By maintaining low viral loads, partners and children would also be protected from horizontal and vertical transmission [31, 32]. Patients would also be protected from the progression to AIDS and associated coinfections. In doing so, we can reduce both mortality and healthcare costs for developing countries [33, 34].

A major downside of relying on CD4^+^ levels to detect treatment failure is the inability to determine the functionality of the T cells being produced. If the patient was co-infected with HTLV (Human T-lymphotropic virus), patients’ CD4^+^ levels could increase, but many of the CD4^+^ cells may actually be nonfunctional [35]. This could camouflage treatment failure, further delaying effective drug regimen switches and lead to a faster progression of AIDS [35].

Historically, there has been resistance to switching to routine viral load testing due to high costs [36]. However, there are cheaper viral load testing options, like the ExaVir^TM^ Load (a simple reverse transcriptase assay), that have the same efficacy as other, more expensive viral load tests [37].

Countries, like Uganda, have successfully switched to using solely viral load testing to determine treatment failure [38]. Countries like South Africa and Thailand have implemented routine viral load testing in addition to the CD4^+^ tests [39–41]. This shows that routine viral load testing is feasible and the WHO should adopt this as the new guideline. We believe that Vietnam and all countries, in general, should follow these steps and update their treatment guidelines to phase out CD4^+^ tests in exchange for viral load testing.

One limitation of our study is the low number of patients with true treatment failure. Also, two different hospital laboratories measured CD4^+^ counts, which could have led to bias in the estimation of immunologic failure. This study did not control for ART treatment during the management of patients. If patients were found to have treatment failure, they were assessed in relation to adherence. If they had good adherence and genotyping showed specific resistance mutations, they were switched to a different treatment regimen. We were also limited to the variables provided in the dataset. For example, the BMI was set at either below and above 18 kg/m^2^. We couldn’t adjust BMI to the cut off for normal and underweight BMI (<18.5 kg/m^2^. Certain variables were only assessed once during the study: BMI at baseline and adherence to ARV after 24 months. This presents a challenge in a causal-relationship type of analysis as we do not have information about how they changed over time. In addition, the findings should be assessed in a prospective study with a larger sample size to further confirm or refute the results.

Finally, we hope our study has shed light on the importance of implementing routine viral load testing as the required test for treatment failure in resource-limited settings.

## Supporting information

S1 Table Original dataset. (XLSX)
